# A study of outcomes, technical safety, and feasibility of D‐2 lymphadenectomy in gastric cancer

**DOI:** 10.1002/jgh3.12402

**Published:** 2020-08-08

**Authors:** Abhijit Talukdar, Srinivas Bannoth, Joydeep Purkayastha, Bibhuti B Borthakur, Deepjyoti Kalita, Gaurav Das, Niju Pegu, Pritesh Singh

**Affiliations:** ^1^ Department of surgical oncology Dr. B. Borooah Cancer Institute Guwahati India

**Keywords:** D‐2 dissection, gastric cancer, lymph node, lymphadenectomy

## Abstract

**Background and Aim:**

Lymph node dissection in gastric cancer had been controversial, but recent data have led us to the conclusion that D‐2 dissection should be the standard of care for potentially curable advanced gastric carcinoma. In this study, we present our single‐institution experience of D‐2 lymph node dissection.

**Methods:**

From January 2013 to September 2018, 115 patients of gastric cancer were treated with D‐2 gastrectomy, 91 of whom met the criteria for study analysis. Data were statistically described as frequencies and percentages where appropriate. Survival curves were plotted using the Kaplan–Meier method, and Cox regression was used to assess the risk among groups. A *P* value <0.05 was considered to be statistically significant at 95% confidence interval.

**Results:**

The majority of patients (86.8%) had Clavien‐Dindo grade I postoperative surgical complications; 90‐day mortality was seen in five (5.5%) patients. Patients with stages I, II, and III had survival rates of 100%, 71.4%; 53.2%, 44.4%; and 27.8%, 28.1%, respectively, for ages <55 and >55 years. Overall recurrence free survival rates were 26 and 28% for <55 years and >55 years, respectively, with a *P* value of 0.570. On multivariate analysis, positive distal margin and multivisceral resection had a statistically significant hazard ratio.

**Conclusions:**

This retrospective study conducted in our institute on patients with gastric cancer undergoing D‐2 lymphadenectomy has shown that the addition of D‐2 lymph node dissection, when performed at high‐volume centers, have acceptable morbidity and mortality rates. This can be seen from our grades of postoperative surgical complications, 90‐day mortality, and overall 5‐year survival.

## Graphical abstract

D‐2 lymph node dissection, when performed at high‐volume centers, have acceptable morbidity and mortality rates as shown by our grades of postoperative surgical complications, 90‐day mortality, and overall 5‐year survival. Multivisceral resection and positive distal margin had a statistically significant hazard ratio.

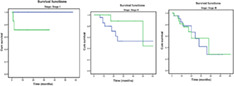

## Introduction

Gastric cancer remains one of the leading causes of cancer‐related death worldwide. Recent advances and a multimodality approach to patients with gastric cancer have led to improved survival compared to the past.^1^ Surgical resection is the main modality in management of gastric cancer patients. As per 2018 GLOBOCON data, gastric cancer constitutes 8.2% of cancer‐related mortality.^2^ Dissection of lymph nodes in gastric cancer has been controversial until recently, but recent data have led us to the conclusion that D‐2 dissection should be the standard of care for potentially curable advanced gastric carcinoma.

Differences in the epidemiology of gastric cancer globally between eastern and western populations and differences in the approach, experience, and outcomes led to this controversy. Surgeons in East Asian countries believe in extended lymphadenectomies for better locoregional control and overall survival; in contrast, surgeons from the west believe that this extended dissection only increases morbidity without significant difference in outcomes.

A recent 15‐year update of a Dutch trial has shown that, when compared to D‐1 surgery, D‐2 lymphadenectomy in gastric cancer is associated with lower locoregional recurrences and better outcomes.^3^

The aim of this study was to present outcomes of D‐2 lymphadenectomy in patients with gastric cancer in our institute.

## Methods

### *Eligibility, staging, and diagnosis*

The data of patients with gastric cancer who underwent D‐2 lymph node dissection, from January 2013 to September 2018, at Dr. B. Borooah cancer institute, Assam, India, were retrospectively analyzed. Institutional scientific and ethical committees approved the study. Ninety‐one patients who underwent D‐2 gastrectomy during this period were identified according to eligibility criteria, and data regarding clinical features, diagnostic and staging workup, and treatment outcomes were collected.

The workup for all patients included a detailed history; physical examination; standard blood tests; contrast‐enhanced computed tomography (CECT) of chest, abdomen, and pelvis; and upper gastrointestinal endoscopy with biopsy. Tumor, node, and metastasis staging were conducted according to the American Joint Committee on Cancer 8th edition for carcinoma of the stomach. Neoadjuvant and adjuvant therapy were administered according to institutional protocol. Patients who did not meet eligibility criteria were excluded from the study.

### *Eligibility criteria*

Patients who had undergone D‐2 lymph node dissection with a minimum number of 16 lymph nodes removed and patients who have been followed up for at least 90 days were included.

### *Outcomes*

The primary outcomes of study were 90‐day mortality, 5‐year overall survival, and recurrence‐free survival. The secondary outcomes were factors effecting overall survival.

### *Grading of postoperative surgical complications and functional status*

The Clavien‐Dindo (CD) classification of surgical complications was used.^4^, ^5^ Functional status of patients was evaluated using the Eastern Cooperative Oncology Group (ECOG) score.^6^

### *Statistical analysis*

Data were statistically described as frequencies (number of cases) and percentages where appropriate. Survival curves were plotted using the Kaplan–Meier method, and Cox regression was used to assess the risk among groups. A *P* value <0.05 was considered to be statistically significant at 95% confidence interval. The statistical analysis was performed using SPSS (statistical package for the social sciences; IBM Corp, Chicago, USA) statistics version 17.0. Overall survival was defined as the time from randomization to death. Recurrence‐free survival was defined as time from randomization to the first recurrence of cancer.

## Results

From January 2013 to September 2018, 115 patients of gastric cancer were treated with D‐2 gastrectomy, 91 of whom met the criteria for study analysis. A summary of demographic, laboratory, and clinical characteristics is presented in Table 1. In brief, of the 91 patients studied, 56 were male, with a mean age of 55.24 (±11.55) years. Preoperative red blood cell transfusion was performed in 27.5%. Diabetes mellitus was seen in 18.7% of patients and hypertension in 15.4%. Gastric outlet obstruction was seen in 12.1%. The majority of patients (71.4%) had ECOG performance status 1.

**Table 1:** Summary of demographic and laboratory characteristics and clinical presentation (*n* = 91)

Characteristics	*n* (%)
Age (years)	
<50	34 (37.4)
>50	57 (62.6)
Gender	
Male	56 (61.5)
Female	35 (38.5)
Mean hemoglobin (g/dL) ± SD	9.20 ± 1.25
Mean albumin (g/dL) ± SD	3.36 ± 0.393
Preoperative transfusion	25 (27.5)
Diabetes mellitus	17 (18.7)
Hypertension	14 (15.4)
Cardiac comorbidity	6 (6.6)
Gastric outlet obstruction	11 (12.1)
ECOG grade	
Grade 1	65 (71.4)
Grade 2	26 (28.6)

ECOG, Eastern Cooperative Oncology Group.

Staging was carried out according to AJCC 8th edition. The majority of patients had stages II (41.8%) and III (47.2%) disease, with the most common (37.4%) histology being well‐differentiated adenocarcinoma (WDAC). According to the site, tumors were divided into five subsites, with the most common site being the antropyloric region (71.4%) (Table 2). Although the majority of our patients had distal tumors, gastric outlet obstruction was seen only in around 12% of patients.

**Table 2:** Summary of clinical and pathology characteristics and surgery (*n =* 91)

Characteristics	*n* (%)
Stage	
I	10 (11)
II	38 (41.8)
III	43 (47.2)
Pathology	
Well‐differentiated adenocarcinoma	34 (37.4)
Moderately differentiated adenocarcinoma	17 (18.7)
Poorly differentiated adenocarcinoma	27 (29.6)
Mucinous adenocarcinoma	5 (5.5)
Signet ring cell carcinoma	8 (8.8)
Site	
Antropyloric	65 (71.4)
Body	4 (4.4)
Body + Antrum	10 (11)
Gastroesophageal junction	2 (2.2)
Body + Gastroesophageal junction	10 (11)
Types of surgery	
Distal gastrectomy	51 (56)
Subtotal gastrectomy	23 (25.3)
Total gastrectomy	15 (16.5)
Esophagogastrectomy	2 (2.2)
Distal pancreatectomy+ splenectomy	1 (0.1)
Mean operative time (hours) ± SD	4.324 ± 1.084
Mean hospital stay (days) ± SD	10.461 ± 4.216

Fifty‐one patients (56%) underwent distal gastrectomy. Subtotal gastrectomy was performed in 23 (25.3%) patients. Fifteen (16.5%) underwent total gastrectomy, and esophagogastrectomy was performed in 2.2% of patients. Distal pancreatectomy along with splenectomy was carried out in one patient. Mean operation time was 4.324 ± 1.084 h. The mean postoperative hospitalization duration of patients was 10.461 ± 4.216 days (Table 2). Proximal margin was free in all patients, and distal margin was positive in four (4.4%) patients. Perinodal spread, lymphovascular invasion, and perineural invasion were seen in 28.6, 26.4, and 11% respectively. The mean lymph node number yield was 20.35 (16–64).

The majority of patients (86.8%) had CD grade I postoperative surgical complications; 90‐day mortality was seen in five (5.5%) of patients, which also included in‐hospital mortality (defined as mortality within 30 days of surgery). Both CD score and 90‐day mortality were low in our study population (Table 3).

**Table 3:** Outcomes of D‐2 dissection (*n* = 91)

Characteristics	*n* (%)	*P‐*value
Clavien‐Dindo grade		
I	79 (86.8)	
II	5 (5.5)	
III	3 (3.3)	
V	4 (4.4)	
90‐day mortality	5 (5.5)	
Stage wise 5‐year overall survival		
Stage I		
<55 years	100	0.335
>55 years	71.4	
Stage II		
<55 years	53.2	0.184
>55 years	44.4	
Stage III		
<55 years	27.8	0.928
>55 years	28.1	
Overall recurrence‐free survival		
<55 years	26	0.570
>55 years	38	

### *Overall survival and recurrence‐free survival*

**Figure 1:**
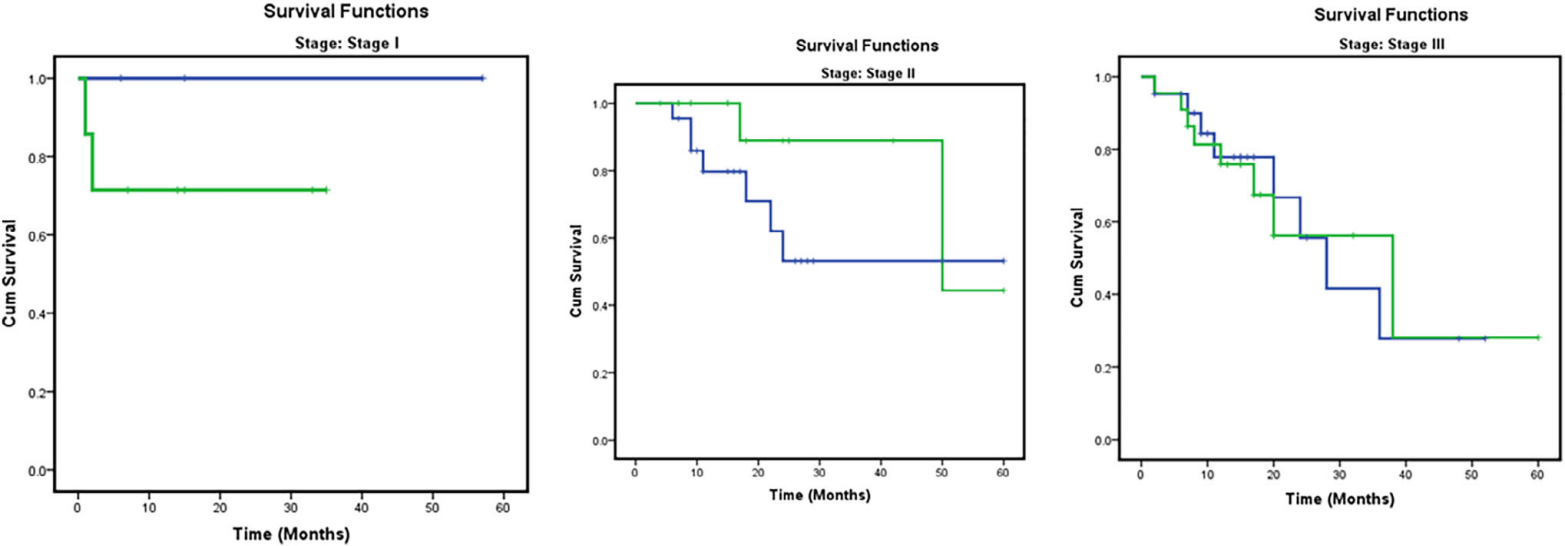
Kaplan–Meier curve for stage‐wise 5‐year survival. Age: (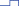), ≤55; (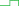), >55; (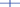), ≤55‐censored; (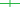), >55‐censored.

**Figure 2:**
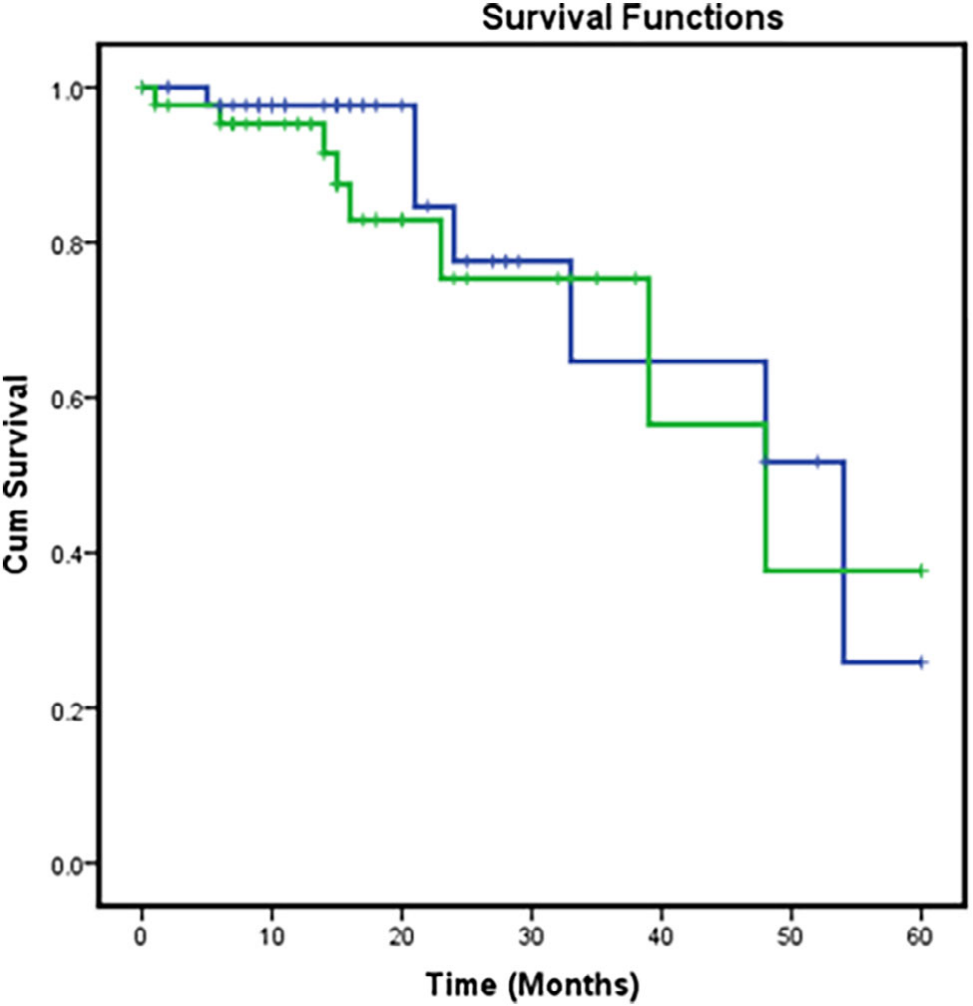
Kaplan–Meier curve for recurrence‐free survival. Age: (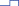), ≤55; (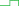), >55; (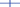), ≤55‐censored; (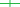), >55‐censored.

Stage‐wise analysis of overall survival was carried out by categorizing patients based on age (<55 and >55 years) as summarized in Table 3. Patients with stages I, II, and III had survival of 100%, 71.4%; 53.2%, 44.4%; and 27.8%, 28.1% with *P* values of 0.335, 0.184, and 0.928, respectively, for ages <55 and >55 years (Table 3, Fig. 1). Overall recurrence‐free survival was 26 and 28% for <55 years and >55 years, respectively, with a *P* value of 0.570 (Table 3, Fig. 2).

### *Univariate and multivariate analysis*

On univariate analysis of variables including age, gender, ECOG status, perinodal spread, Lympho‐vascular invasion (LVI), Peri‐neural invasion (PNI), multivisceral resection, and positive distal margin, patients with multivisceral resection (hazard ratio [HR] 7.26, 0.937–56.282, *P* = 0.058) and positive distal margin (HR 3.075, 1.033–9.153, *P* = 0.043) had significant HRs (Table 4). On multivariate analysis (Table 5), positive distal margin (HR 3.175, 1.062–9.494, *P* = 0.039) and multivisceral resection (HR 7.193, 1.016–61.596, *P* = 0.048) had statistically significant HRs.

**Table 4:** Univariate analysis (*n* = 91)

Characteristic	HR (95% CI)	*P*‐value
Age > 50 years	0.978 (0.453–2.11)	0.955
Gender: Female	1.173 (0.541–2.545)	0.686
ECOG status	1.412 (0.613–3.254)	0.417
Perinodal spread	2.077 (0.974–4.427)	0.58
PNI	1.75 (0.604–5.080)	0.303
LVI	1.100 (0.464–2.612)	0.828
Multivisceral resection	7.26 (0.937–56.282)	0.058
Positive distal margin	3.075 (1.033–9.153)	0.043

CI, confidence interval; ECOG, Eastern Cooperative Oncology Group; HR, hazard ratio; LVI, Lympho‐vascular invasion; PNI, Peri‐neural invasion.

**Table 5:** Multivariate analysis (*n* = 91)

Characteristic	HR (95% CI)	*P*‐value
Positive distal margin	3.175 (1.062–9.494)	0.039
Multivisceral resection	7.913 (1.016–61.596)	0.048

CI, confidence interval; HR, hazard ratio.

### *Complications*

Postoperative complications include duodenal blowout syndrome (4.4%), surgical site infection (8.8%), chyluria (2.2%), deep vein thrombosis (1.1%), gastrointestinal bleed (1.1%), burst abdomen (2.2%), lower respiratory tract infection (4.4%), and perifeeding jejunostomy bile leak (2.2%).

Around nine (9.9%) patients had locoregional recurrence, and hepatic metastasis was seen in five (5.5%). Pulmonary metastasis and ovarian and skin metastasis were seen in two (2.2%), one (1.1%), and two (2.2%) patients, respectively.

## Discussion

Carcinoma of the stomach remains one of major causes of cancer‐related death. Gastric cancer is more common in old age, which usually occurs in the sixth to seventh decade of life, with mean age of incidence approximately 67 years.^7^ Gastric carcinoma was historically predominantly of the distal type, but recently, there have been declining trends of the distal tumor with an increase in the rates of proximal cardia tumors.^8^

The rationale for extensive lymph node dissection is that there is a better oncological outcome, but a point of concern is that this oncological benefit should surpass the postoperative morbidity and mortality associated with these extensive dissections.^9^ Extensive lymphadenectomy serves three purposes: staging of disease, better locoregional control, and increased overall survival. A minimum of 16 lymph nodes should be examined according to the staging manual of American Joint Committee on Cancer.^10^

Differences in the epidemiology of gastric cancer, surgical approaches, and experience between western and eastern scenarios has led to controversy over the extent of lymphadenectomy in the treatment of gastric cancer for decades, until the recent publication of the 15‐year update of a Dutch trial, which definitely showed the superiority of D‐2 dissection for locoregional control and overall outcome. The basis of the difference in opinion between the western hemisphere and eastern hemisphere on D‐2 lymphadenectomy comes from two randomized controlled trials, one Dutch trial from Netherlands and a United Kingdom Medical Research Council trial. Both these trials were of the opinion that in‐hospital mortality was significantly higher in the D‐2 lymphadenectomy group compared to the D‐1 group, with no additional benefit in overall survival at 5 years. In both these trials, distal pancreatectomy and splenectomy were routinely performed as a part of D‐2 lymph node dissection. Results of these trials can no longer be accepted in the current practice scenario as there is no benefit of the routine resection of distal pancreas and spleen. Spleen‐ and pancreas‐preserving D‐2 lymphadenectomy improves overall survival, with acceptable perioperative morbidity and mortality.^11^, ^12^, ^13^, ^14^, ^15^, ^16^, ^17^, ^18^

Outcomes of D‐2 lymphadenectomy are influenced by surgical skills and the volume of center. Japanese centers had a high volume of cases and relatively better surgical skills and experience. So, this difference of opinion between the west and Japan could be explained as surgeons from the west who took part in the trials having very little experience with D‐2 gastrectomy and its outcomes.^19^ For D‐2 resection with acceptable morbidity and mortality, it requires a steep learning curve, and a surgeon is expected to perform 30–40 D‐2 lymphadenectomies.^20^, ^21^, ^22^, ^23^, ^24^ Randomized trials from Taiwan and Italy have shown that patients who undergo D‐2 resections in high‐volume centers had better survival, with an acceptable mortality rate of around 3%.^25^, ^26^

Our institute is a tertiary cancer referral center for the entire northeast part of India, with a major bulk of gastrointestinal and hepatobiliary cancers. Data from our institute also highlight that D‐2 gastrectomy is technically safe and feasible with accepted mortality and morbidity rates when performed at centers with expertise, as seen in randomized trials from Taiwan and Italy.^25^, ^26^ Postoperative surgical complications in our study population as indicated by CD grades show that spleen‐ and pancreas‐preserving D‐2 lymphadenectomy was associated with acceptable morbidity. Most of our patients (86.5%) had CD grade I complications, which were managed with simple conservative measures. Only 4.4% of patients had a CD score of grade V, and 90‐day mortality of 5.5% in our study may be acceptable for gastric cancer surgery. Patient in whom distal pancreatectomy with splenectomy was performed expired within 5 months of surgery.

Nasojejunal feeding is preferred at our institute for postoperative enteral nutrition. Feeding jejunostomy was performed only in 27 (29.7%) patients. Feeding was started routinely from postoperative day 1 unless contraindicated. Two patients had perifeeding jejunostomy tube bile leak, which was conservatively managed.

When stage‐wise analysis was conducted, patients younger than 55 years of age had better overall survival. Patients with stages I, II, and III had survival of 100%, 71.4%;, 53.2%, 44.4%; and 27.8%, 28.1%, respectively, for ages <55 years and >55 years. Patients with positive distal margin and multivisceral resection had statistically significant HRs.

The advantages of our study were that surgeries had been performed by surgical oncologists who were trained at high‐volume centers for gastric cancer, which is one of the important factors for the outcomes of this radical procedure. Data of patients in whom fewer than 16 lymph nodes were harvested were excluded from analysis. Disadvantages of this study are that an analysis of the impact of perioperative chemotherapy on outcomes could not be carried out.

In conclusion, this retrospective study from our institute on patients with gastric cancer undergoing D‐2 lymphadenectomy has shown that the addition of D‐2 lymph node dissection, when performed at high‐volume centers, has acceptable morbidity and mortality rates. This can be seen from our grades of postoperative surgical complications, 90‐day mortality, and overall 5‐year survival. Positive distal margin and multivisceral resection had statistically significant HRs.
